# 19q13.33→qter trisomy in a girl with intellectual impairment and seizures

**DOI:** 10.1016/j.mgene.2014.09.004

**Published:** 2014-10-27

**Authors:** Gianna Carvalheira, Mariana Moysés Oliveira, Sylvia Takeno, Fernanda Teresa de Lima, Vera Ayres Meloni, Maria Isabel Melaragno

**Affiliations:** a Universidade Federal de São Paulo — UNIFESP, Department of Morphology and Genetics, São Paulo, Brazil; b Universidade Federal de São Paulo — UNIFESP, Department of Mastology, São Paulo, Brazil

**Keywords:** Translocation t(19q;21p), *de novo* 19q13.33 trisomy, Gene regulation, Intellectual impairment, Seizures

## Abstract

Rearrangements in chromosome 19 are rare. Among the 35 patients with partial 19q trisomy described, only six have a breakpoint defined by array. The 19q duplication results in a variable phenotype, including dysmorphisms, intellectual disability and seizure. In a female patient, although G-banding at 550 band-resolution was normal, multiplex ligation-dependent probe amplification (MLPA) technique and genomic array showed a 10.6 Mb terminal duplication of chromosome 19q13. Fluorescent *in situ* hybridization (FISH) revealed that the duplicated region was attached to the short arm of chromosome 21 and silver staining showed four small acrocentrics with nucleolar organization region (NOR) activity, suggesting that the breakpoint in chromosome 21 was at p13. This is the first *de novo* translocation between 19q13.33 and 21p13 described in liveborn. The chromosome 19 is known to be rich in coding and non-coding regions, and chromosomal rearrangements involving this chromosome are very harmful. Furthermore, the 19q13.33→qter region is dense in pseudogenes and microRNAs, which are potent regulators of gene expression. The trisomic level of this region may contribute to deregulation of global gene expression, and consequently, may lead to abnormal development on the carriers of these rearrangements.

## Highlights

• The first patient with a *de novo* translocation between 19q13.33 and 21p13 reported in liveborn.
• The patient clinical and cytogenetic analyses are reported in details.
• Rearrangements in 19q13.33→qter region are correlated to intellectual disability and seizures.
• Chromosomal rearrangements involving rich coding and non-coding regions appear to be very harmful.

## Introduction

Developmental delay and intellectual disability affect around 3% of the general population (Shevell et al., 2003). In many cases, although patients present normal karyotypes, they can be carriers of cryptic genomic imbalances which, when detected, are important for both accurate diagnosis and genetic counseling. Rearrangements involving chromosome 19, either duplications or deletions, have rarely been reported. The main clinical features in partial 19q trisomy include low birth weight, short stature, abnormal ears, short neck, intellectual disability and seizures (Dorn et al., 2001, Lenzini et al., 2010). Seven patients with pure 19q trisomy, and 28 patients with other concomitant chromosome imbalances, have been published. We describe a female patient, carrier of a *de novo* terminal 19q trisomy, the first case of translocation between 19q and 21p in liveborn. The extra 19q region contains a high density coding and non-coding DNA sequences, including both pseudogenes and microRNAs, which have a critical role in gene expression control. The deregulation of transcript levels due to the chromosomal rearrangement may lead to abnormal development and other clinical features. Here, three specific genes and the possible roles of regulatory elements, at trisomic level, in the 19q region will be discussed, as well as the relationship between phenotype and possible molecular mechanisms involved in the clinical characteristics of the patient.

## Patient and methods

### Clinical report

The patient described here, an only child of a young healthy and consanguineous couple, presents with mild dysmorphic features and intellectual disability (Table 1). The female patient was born at term by vaginal delivery, with a weight of 2900 g (50th centile), and unreported length and head circumference. At eight days old, she was hospitalized with a urinary tract infection. Until the age of four years, she had other urinary tract infections, bronchopneumonia episodes, urolithiasis and anemia. She evolved with moderate neuromotor developmental and speech delay, and non-quantified intellectual disability. At the age of four years she started to have seizures, which have been controlled by valproic acid and levomepromazine treatment to date. She is now aged 12 years. Upon genetic evaluation, at eight years and 10 months of age, her measurements were: height 112 cm (< 3rd centile), weight 23 kg (10th–25th centile), and head circumference 51 cm (50th centile). Her main dysmorphic features were (Supplementary data, Fig. S1): short stature; ocular hypertelorism; downturned corners of mouth; posteriorly rotated ears; prominent antihelix; short neck; short, cold and congested hands and fingers; clinodactyly of the 5th fingers; and thoracolumbar scoliosis. Because of the congested hands she was referred to a rheumatologist for evaluation, which was normal.

### Cytogenetic and molecular analysis

Chromosomal analysis with 550 resolution G-banding was performed on lymphocyte cultures from the patient and her parents, based on a total of 20 metaphase cells. Genomic DNA from whole blood was isolated using the Gentra Puregene Kit (Qiagen-Sciences, Germantown, USA). MLPA (multiplex ligation-dependent probe amplification) technique, using the SALSA MLPA P070 Human Telomere-5 kit (MRC-Holland, Amsterdam, The Netherlands), that contains probes for subtelomeric regions, was performed to verify possible cryptic genome imbalances. Genomic array was performed using the Affymetrix Genome-Wide Human SNP Array 6.0 (Affymetrix Inc., Santa Clara, CA, USA), and the data were analyzed with the Genotyping Console 3.0.2 and Chromosome Analysis Suite (ChAS) software (Affymetrix) based on GRCh37/hg 19. To validate the results from MLPA and array assays, and to investigate the trisomic segment localization, fluorescent *in situ* hybridization (FISH) on metaphase spreads was performed using a BAC probe for 19q13.43 (RP11-359B7). Silver staining was performed to verify the nucleolus organizer region (NOR) activity, using 50% silver nitrate in formic acid water.

## Results

**Fig. 1:**
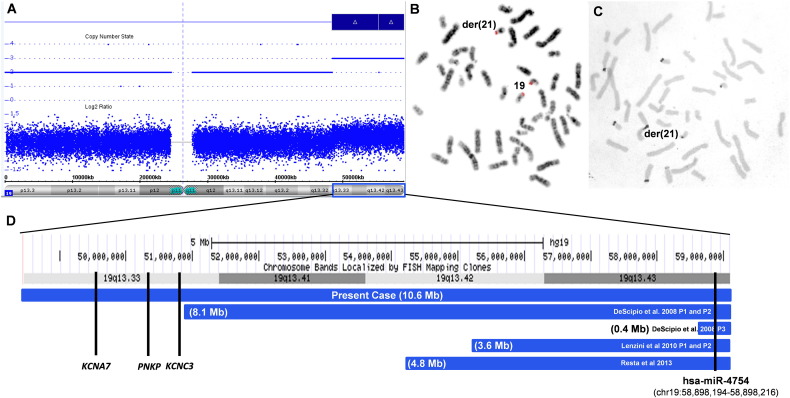
(A) Array profile showing three copies of 19q13.33q13.43 (blue bar). (B) FISH inverted DAPI-banding, in metaphase chromosomes, using RP11-359B7 probe at 19q13.43, showing two signals in chromosomes 19 and one signal in the short arm of the derivative chromosome 21. (C) Silver staining showing four small acrocentric chromosomes with nucleolar activity, including the der(21). (D) Schematic representation of the chr19:48,463,121-59,097,842 region presented in trisomy in our patient, showing *KCNA7*, *PNKP*, *KCNC3* genes at the 19q13.33 band. The blue lines represent the array results from the six patients previously described, with their respective three copies segment sizes. In 19q13.43, the location of miR-4754 *locus* mapped at chr19:58,898,194-58,898,216 is shown.

G-banding karyotypes of the patient (Supplementary data, Fig. S2A) and her parents were normal. Due to phenotypic features present in the patient, MLPA was performed and revealed three copies of the subtelomeric 19q region with probe *BC-2*, localized in 19q13.43 (Supplementary data, Fig. S2B and S2C). Genomic array showed a 10.6 Mb triplication of 19q as follows: arr19q13.33q13.43(48,463,121-59,097,842)×3 (Fig. 1A). Since the array technique does not allow the determination of the extra segment position, FISH with a 19q13.43 BAC probe (RP11-359B7) revealed that it was attached to the short arm of one chromosome 21 in the patient (Fig. 1B) and showed two normal signals for both parents (Supplementary data, Fig. S2D and S2E), indicating a *de novo* unbalanced translocation. The silver staining revealed that four small acrocentrics presented NOR activity (Fig. 1C), revealing that the der(21) has active NOR and therefore the breakpoint was mapped at the 21p13 band. Therefore, the patient's final karyotype is 46,XX,der(21)t(19;21)(q13.33;p13)dn.

## Discussion

Chromosomal alterations are rare and usually incompatible with life. It has been described that 4.2% of the chromosomal abnormalities found in miscarriages involve 19q, most of them in trisomic state and only one case presenting monosomy (Azmanov et al., 2007). In fact, the chromosome 19, which is one of the smallest human chromosomes, is the third in number of genes, making its imbalance more damaging (Grover et al., 2004).

To date, 35 cases have been described with partial distal 19q trisomies, most of them associated with imbalances of other chromosome regions, due to familial balanced translocations or *de novo* unbalanced translocations (Table 1S). It is not precisely known which genes are responsible for the phenotype in patients with 19q13.3→qter trisomy. Our patient shares some clinical features with other carriers of partial 19q trisomy, especially those described by Dorn et al. (2001) and Lenzini et al. (2010), who present short stature, abnormal ears, short neck, intellectual disability, motor developmental delay, speech delay and seizures (Table 1). Dorn et al. (2001) discussed the different mechanisms involved in the epileptic phenomena. They drew attention to the photoparoxysmal response that is a symptom of photosensitivity and may constitute a genetic trait, which is also found in myoclonic epilepsy in infants (benign or severe) or myoclonic astatic epilepsy in early childhood. In 19q13.3 region there are two genes (*KCNC3* and *KCNA7*) associated with the potassium channel that could be related to seizures. Furthermore, recessive mutations have been reported in *PNKP* gene that cause epileptic encephalopathy in early infancy (Fig. 1D), although the effects of these three genes at trisomic level are yet unknown. Additionally, the Specific Language Impairment (SLI) Consortium identified a 19q linkage region to speech delay or dysfunction (Consortium, 2004), as mentioned in eight patients described with partial 19q trisomy (Table 1 and Table 1S).

Among the 35 patients with partial 19q trisomy reported in literature, 12 have pure partial 19q trisomy, resulting from translocations involving the short arm of acrocentric chromosomes (Table 1S). However, this is the second description of a patient with a pure 19q trisomy attached to the short arm of chromosome 21 (Babic et al., 2007). It has been proposed that in interphase nucleus, the close proximity of chromosome domains is a rearrangement predisposition factor between chromosome 19 and the short arm of acrocentric chromosomes (Valerio et al., 1993). It has been reported that repetitive sequences, like *Alu* elements (Grover et al., 2003, Grover et al., 2004), are present in 19q region (Cotter et al., 1997) and also that the acrocentric short arms are rich in β-satellite and satellite III families (Bandyopadhyay et al., 2001). Even though chromosome 21 has few *Alu* sequences (Grover et al., 2003), it is possible that these few *Alu* elements may eventually recombine to the 19q region, which is dense in repetitive DNA (see Supplementary data, Fig. S3).

Familial acrocentric short arm heteromorphisms could also occur in some patients (Benzacken et al., 2001, Natiq et al., 2014, Starke et al., 2005, Trifonov et al., 2003). However, in our patient, this hypothesis was discarded due to two reasons: first, all the acrocentric short arms look normal at the 550 resolution G-banding, and second, the SNP array results showed that the presence of 19q13 in trisomic level, as well as FISH results confirmed the extra 19q13 region was localized in one of the chromosome 21 short arms. These findings confirm that this unbalance rearrangement was in fact a cryptic chromosomal alteration instead acrocentric short arm heteromorphism.

Considering that hundreds of non-coding sequences are mapped in the high density genic 19q13.33→qter region, including 112 pseudogenes and 76 microRNA *loci*, the presence of these non-coding sequences can give important insights into the evolutionary history of a genomic region in terms of regulatory roles of these sequences. Many of these pseudogenes, observed in this extra 19q region, are adjacent to their cousin coding genes *loci*. It is known that the pseudogenes can compete for positive or negative stabilizing factors and/or microRNAs, altering the levels of mRNAs that would be translated in a specific developmental stage or tissue. This class of regulatory elements is called “competing endogenous RNA” (ceRNA), in which pseudogenes belong. These ceRNAs act as microRNA “sponges” by sharing common miRs sites with their mRNA cousins, inhibiting normal miRs activity (Pink et al., 2011, Sen et al., 2014, Tay et al., 2014). In the same way, the large amount of pseudogenes and microRNAs sequences in the 19q13.33→qter region suggests a regulatory function and both of them have emerged as regulatory elements of gene expression (Pink et al., 2011, Sen et al., 2014). Under these assumptions, any chromosomal rearrangement in this region can alter the expression level of many genes by interfering in mRNAs translation. In the patient described herein, the 19q13.33→qter region is in trisomy and it is possible that both pseudogenes and microRNAs are highly expressed, changing the normal homeostasis and cell context, consequently interfering with her phenotype.

Among the 35 patients with 19q related trisomy, only six with 19q13.3→qter in three copies were analyzed by high resolution CGH- or SNP-array, for a precise breakpoint determination (Table 1). The main clinical features shared by these patients are short neck, intellectual disability, motor developmental delay and speech delay (Table 1). In the minor 0.4 Mb overlapping region, mapped at the 19q13.4 band, there are 26 *loci*: 18 coding genes, four pseudogenes, two microRNAs and two uncharacterized *loci* (Table 2S). The 18 coding genes were involved in many biological processes, not being correlated with a specific phenotype. On the other hand, one of the microRNAs (has-miR-4754, Fig. 1D) showed, by *in silico* analysis using three different algorithms (miRDB (http://mirdb.org/miRDB/), TargetScan (http://www.targetscan.org/vert_40/), and TargetMiner (http://www.isical.ac.in/~bioinfo_miu/targetminer20.htm)), six possible target genes: *EYA4*, *S1PR3*, *ANGPTL2*, *CNTN3*, *JMY* and *PPP1R16*. These genes appear to be related to central nervous system development and brain function, kidney development and disease, as well as vascuologenesis (Firat-Karalar et al., 2011, Meng and Lee, 2009, Richardson et al., 2014, Shimoda and Watanabe, 2009). It is plausible to speculate that the miR-4754 is upregulated in these six patients, due to the trisomic level of the 19q13.4, resulting in these six target genes having their mRNAs and proteins depleted, adding to the patient's phenotype. All these hypotheses need to be proven. The microRNA functional mechanism was first associated with chromosomal alterations in a patient with trisomy 21. The miR-155, mapped at 21q21.3, targets *AGTR1* gene (angiotensin II receptor, type 1), and is highly expressed in trisomic cells resulting in a decreased amount of the AGTR1 protein. AGTR1 has been associated with hypertension, leading to a hypothesis that lower blood pressure in patients with trisomy 21 could be partially caused by the overexpression of miR-155 (Sethupathy et al., 2007). Therefore, the comprehension of the molecular mechanisms that occur between chromosomal rearrangements and gene expression control is fundamental to establish the phenotype/genotype correlation as well as to understand the normal biological development.

## Contributorship statement

Gianna Carvalheira performed silver staining and *in silico* analysis as well as wrote the paper; Mariana Oliveira performed the MLPA and SNP array as well as analyzed these results; Sylvia Takeno performed the cytogenetic and FISH analysis, Fernanda Lima and Vera Ayres Meloni carried out the data clinical of the patient, finally Gianna Carvalheira and Maria Isabel Melaragno coordinated the study. All authors read and approved the final manuscript.

## Web resources

The URLs for data presented herein are as follows:

MiRDB: http://mirdb.org/miRDB/
TargetScan: http://www.targetscan.org/vert_40/
TargetMiner: http://www.isical.ac.in/~bioinfo_miu/targetminer20.htm

## Figures and Tables

**Table 1:** Clinical features: Comparison of the present case with both cases described by Dorn et al. (2001) and the cases analyzed by array.

Patients	Dorn et al. (2001) P1	Dorn et al. (2001) P2	DeScipio et al. (2008) P1	DeScipio et al. (2008) P2	DeScipio et al. (2008) P3	Lenzini et al. (2010) P1	Lenzini et al. (2010) P2	Resta et al. (2013) P1	Present case
Method of detection	G-banding	G-banding	array CGH^a^	array CGH^a^	array CGH^a^	SNP array^a^	SNP array^a^	array CGH^a^	SNP array/FISH
Proximal 19q breakpoint	19q13.3	19q13.3	19q13.33	19q13.33	19q13.43	19q13.42	19q13.42	19q13.42	19q13.33
Triplication size (Mb)			8.1	8.1	0.4	3.6	3.6	4.8	10.6
de novo	−	−	NI	−	NI	−	−	+	+
Sex	M	F	M	F	M	M	M	F	F
Age	34y	31y	NI	NI	NI	5y	49y	1y	8y
Short stature	+	+	+	+	NI	−	+	NI	+
IUGR/growth delay	+/+	+/−	NI	NI/+	NI	−/−	−/−	−/−	−/+

*Facial features*									
Frontal bossing	−	−	NI	NI	NI	+	−	NI	−
Ocular hypertelorism	−	−	NI	NI	NI	+	−	+	+
Nasal root abnormalities	+	+	NI	NI	NI	+	+	NI	−
Abnormal ears	+	+	NI	NI	NI	+	−	NI	+
Downturned corners of the mouth	−	−	NI	NI	NI	−	−	NI	+
Small teeth with dystrophic enamel	−	−	NI	NI	NI	+	−	NI	−
Short neck	+	−	NI	NI	NI	+	+	NI	+

*Hands and feet*									
Joint hyperlaxity	−	−	NI	NI	NI	+	−	NI	−
Tapering fingers	−	−	NI	NI	NI	+	−	NI	+
Brachydactly	−	−	NI	NI	NI	+	−	NI	+
Bilateral clinodactyly	−	−	NI	NI	NI	−	+	+	+
Congested hands	NI	NI	NI	NI	NI	NI	NI	NI	+

*Nervous system*									
Intellectual disability	+	+	NI	NI	NI	+	+	+	+
Motor developmental delay	+	+	NI	+	NI	+	+	+	+
Speech delay	+	+	NI	NI	NI	+	+	NI	+
Seizures	+	+	NI	NI	NI	−	+	+	+
Structural brain abnormalities	−	−	NI	NI	NI	−	NI	+	NV
Hypoplasia of corpus callosum	−	−	NI	NI	NI	−	NI	+	NV
Hypotonia	−	−	NI	NI	NI	+	−	+	−

*Other*									
Pulmonary infections	−	−	NI	NI	NI	−	−	NI	+
Urinary tract infections	+	−	NI	NI	NI	−	−	NI	+
Urolithiasis	−	−	NI	NI	NI	−	−	NI	+
Bilateral nystagmus	+	+	NI	NI	NI	NI	NI	+	−
Growth hormone deficiency	−	−	+	NI	NI	−	−	−	−
Hypothyroidism	−	−	+	NI	NI	−	−	−	−

P: Patient; -: No; +: Yes; y: Years; NI: Not informed; NV: Not verified.

a All the breakpoint coordinates were converted to GRCh37/hg19.
