# Supplementary material for: 19q13.33→qter trisomy in a girl with intellectual impairment and seizures
Source: Meta Gene. 2014 Oct 27;2:799–806. doi: 10.1016/j.mgene.2014.09.004 (PMC4288793; doi:10.1016/j.mgene.2014.09.004)
Supplement: Supplementary file 1 — Supplementary material. [file mmc1.pdf]

Legends of supplementary figures:

Fig. S1. (A) Patient at 11 years-old showing ocular hypertelorism, downturned corners of mouth, posteriorly rotated ears and prominent antihelix. (B) Short and congested hands with clinodactyly of the 5th fingers.

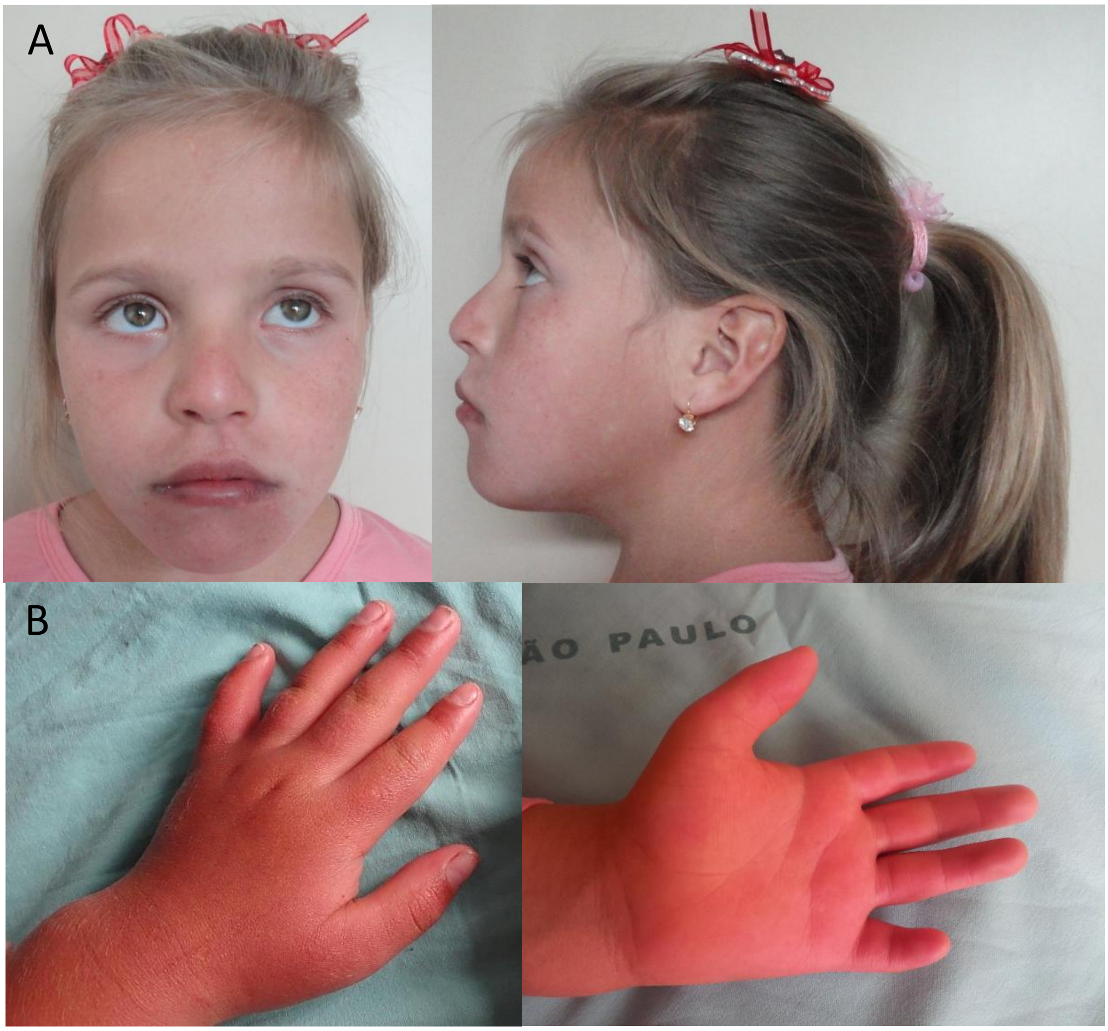

Fig. S2. (A) Normal karyotype 46,XX, using G-banding at 550 band-resolution. (B) MLPA analysis graphic, using SALSA MLPA kit P070 Human Telomere-5, showing three copies of BC-2 probe, located on 19q subtelomeric region (red dot). (C) Table showing the MLPA peak ratios of the BC-2 probe (1.482), indicating a trisomy of this region. (D, E) FISH inverted DAPI-banding, in metaphase chromosomes, using RP11-359B7 probe at 19q13.43, showing two signals at chromosomes 19 on both parents.

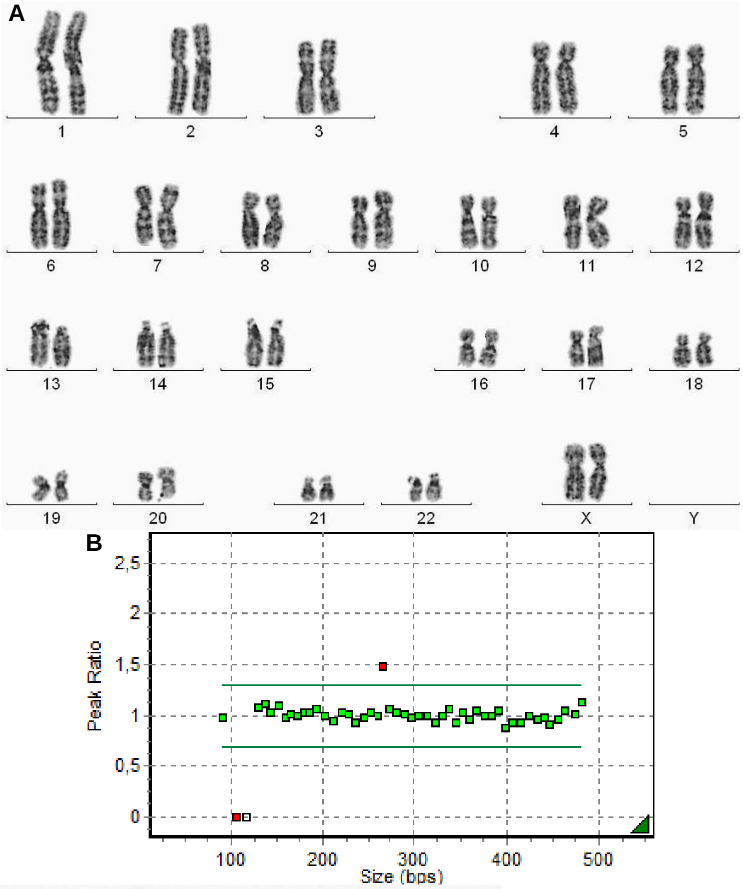

C

	Probe Name	Bin Size	35B_001_A05
1	92	92.2	0.987
2	UTY	106.3	0.000
3	DBY	118.1	-1
4	KIAA1720	130.2	1.089
5	APG4B	137.2	1.108
6	KIAA0226	143.8	1.024
7	FRG1	151.8	1.096
8	GNB2L1	159.6	0.980
9	TBP	166.0	1.012
10	VIPR2	172.4	1.003
11	RECQL4	179.4	1.030
12	EU-HMTase1	186.4	1.027
13	ECHS1	193.3	1.056
14	KIAA1030	201.5	0.997
15	ZNF10	211.4	0.943
16	CDC16	219.7	1.038
17	MTA1	227.6	1.006
18	FLJ22604	235.5	0.925
19	GAS11	243.9	0.973
20	SECTM1	252.1	1.034
21	CTDP1	260.7	0.989
22	BC-2	266.9	1.482
23	FLJ20517	274.1	1.057
24	S100B	281.0	1.034
25	ARSA	290.2	1.019
26	SYBL1	297.6	0.972
27	TNFRSF18	305.8	0.993
28	ACP1	314.4	0.993
29	CHL1	323.3	0.926
30	ZNF141	330.5	1.000
31	LOC133957	338.9	1.072
32	IRF4	345.5	0.931
33	UNC84A	353.7	1.023
34	FBXO25	360.5	0.962
35	FLJ00026	368.4	1.048
36	BS69	377.0	0.989
37	BET1L	384.4	0.998
38	RBBP2	391.5	1.040
39	PSPC1	399.8	0.874
40	ADPRTL2	406.4	0.936
41	NDN	416.0	0.930
42	DECR2	423.9	1.001
43	RPH3AL	433.8	0.961
44	THOC1	441.3	0.987
45	PPAP2C	446.8	0.910
46	FLJ22115	455.3	0.957
47	STCH	462.6	1.042
48	IL17R	475.0	1.021
49	SHOX	481.4	1.125

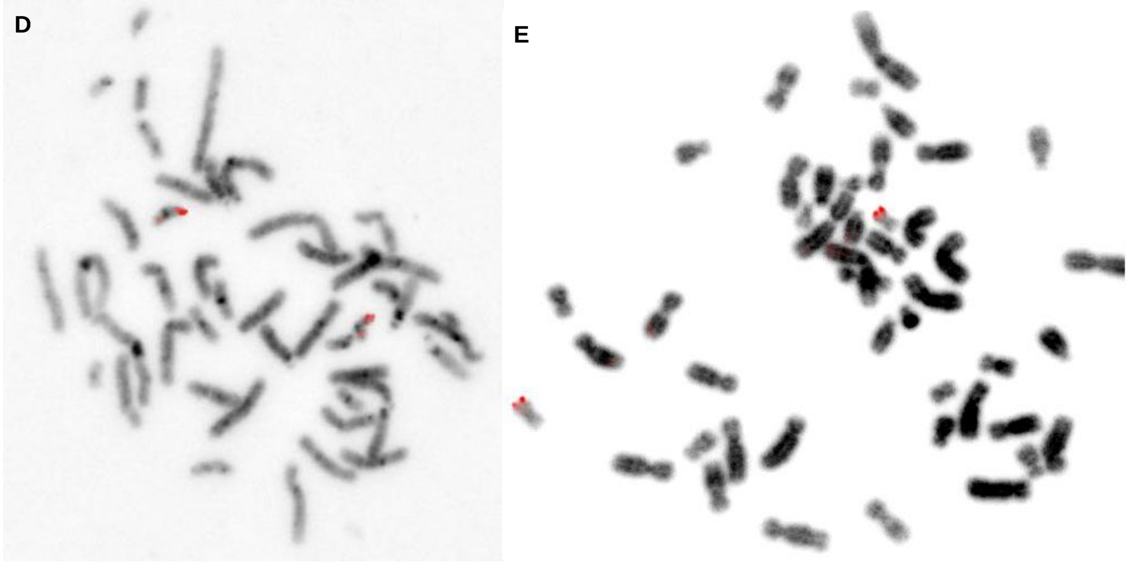

Fig. S3. (A) Array profile showing 19q13.33 in triplication (blue bar*).* (B) Schematic representation of the chr19:48,363,121-48,563,121 region (100 kb upstream and downstream of the breakpoint at 19q13.33), showing SINEs, LINEs, LTRs and others repetitive sequences present in this region.

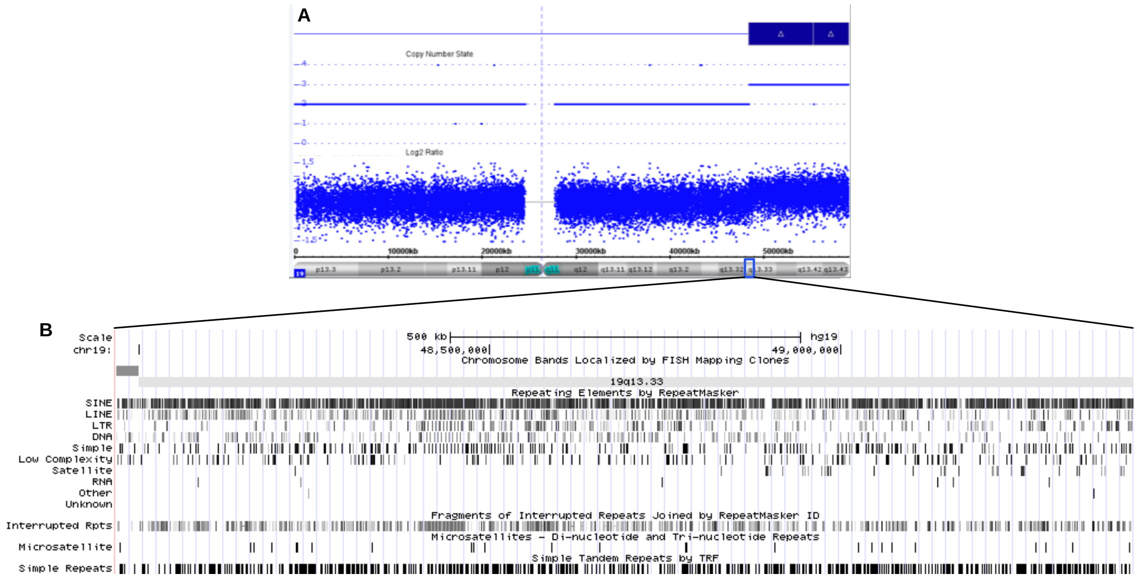

Table 1S Clinical features described on the literature.

	Lange and Alfi (1976)	Schimid (1979)	Zonana et al (1982)	Rivas et al (1985)	Boyd et al (1992)	Quack et al (1992)
**PATIENTS**	2	2	2	1	1	1
**Proximal 19q breakpoint described by authors**	**t(19;22)(q13;p13)mat**	**t(19;20)(q133;20pter)mat**	**t(19;?)mat**	**46,XX,-22,+der(22), t(19;22)(q13.2;p11)**	**46,XY,-22,+der(22),t(19;22)(q13.33;p11.2)mat**	**r(19)**
de novo	N	N	N	Y	N	N
**Sex**	NI	male/female	male	female	male	male
**Age**	NI	12y/9y		2y4m	1y8m	
**Method of detection**	NI	G-banding	G-banding	G-banding	NI	FISH
**Short stature**	NI	Y/NI	NI	Y	NI	NI
**IUGR/Growth delay**	NI/Y	Y/Y	Y/Y	Y/Y	NI/Y	NI
**FACIAL FEATURES**						
**Ocular hypertelorism**	Y	Y/	NI	NI	NI	Y
**Nasal root abnormalities**	Y	Y/Y	Y	Y	NI	Y
**Abnormal ears**	NI	Y/Y	NI	Y	NI	NI
**Downturned corners of the mouth**	Y	Y/Y	Y	Y	NI	Y
**Small teeth with dystrophic enamel**	NI	Y/Y	NI	NI	NI	NI
**Short neck**	NI	Y/Y	NI	NI		NI
**HANDS AND FEET**						
**Bilateral clinodactyly**	NI	N	NI	NI	NI	NI
**NERVOUS SYSTEM**						
**Intellectual disability**	Y	Y/Y	Y	Y	Y	Y
**Motor developmental delay**	Y	Y/Y	Y	Y	Y	Y
**Speech delay**	NI	Y/	NI	NI	NI	NI
**Seizures**	Y	N/Y	Y	N	Y	NI

**Structural brain abnormalities**	NI	Y/Y	NI	N	NI	NI
**Hypoplasia of Corpus Callosum**	NI	N	NI	N	NI	NI
**Hypotonia**	NI	Y/Y	NI	Y	NI	
**OTHER**						
**Pulmonary infections**	NI	N	NI	N	NI	NI
**Urinary tract infections**	NI	Y/N	NI	N	NI	NI

NA: Not Applicable; **N:** no; **Y:** yes; **y:** Years; **m: months; NI:** Not Informed.

Table 1S Clinical features described on the literature. (continuation)

	Trautmann et al (1993)	Valerio et al (1993)	James et al (1996)	Cotter et al (1997)	Bhat et al (2000)	Tercanli et al (2000)
**PATIENTS**	1	1	1	1	1	1
**Proximal 19q breakpoint described by authors**	**46,XX,del(16)(q13.08-21.05),dup(19)(q13.13-13.2)**	**t(13;19)(p13;q13.2)mat**	**46,XX,-10,+der(10)t(10;19)(q26.3;q13.3)**	**46,XY,dir dup(19)(q13.2q13.4)dn**	**46,XY,dup(19)(q13.4 q13.3)dn**	**46,XY,dup(19)(q13.1→qter)dn**
de novo	Y	N	Y	Y	Y	Y
**Sex**	female	male	female	male	male	male
**Age**	newborn	died at 10 days	3y6m	-	1y6m	-
**Method of detection**	G-banding	G-banding	FISH	G-binding in chorionic villus/wpc19	wpc19	G-banding in chorionic villus
**Short stature**	NI	NA	Y	NA	Y	NA
**IUGR/Growth delay**	N/Y	N/NA	Y/Y	NA	Y/Y	Y/NA
**FACIAL FEATURES**						
**Ocular hypertelorism**	NI	NI	NA	NA	Y	NA
**Nasal root abnormalities**	NI	Y	Y	NI	Y	NI
**Abnormal ears**	NI	Y	Y	NI	Y	NI
**Downturned corners of the mouth**	NI	NI	Y	NI	Y	NI
**Small teeth with dystrophic enamel**	NA	NA	NI	NA	NI	NA
**Short neck**	NI	NI	Y	NI	Y	NI
**HANDS AND FEET**						
**Bilateral clinodactyly**	NI	Y	Y	NI	NI	NI
**NERVOUS SYSTEM**						
**Intellectual disability**	Y	NA	Y	NA	Y	NA
**Motor developmental delay**	Y	NA	Y	NA	Y	NA
**Speech delay**	NA	NA	Y	NA	NI	NA

**Seizures**	NI	N	N	NA	Y	NA
**Structural brain abnormalities**	N	Y	N	NI	NI	N
**Hypoplasia of Corpus Callosum**	N	Y	N	NI	NI	N
**Hypotonia**		Y	N	NA	NI	NA
**OTHER**						
**Pulmonary infections**	Y	NI	N	NA	Y	NA
**Urinary tract infections**	NI	NI	N	NA	NI	NA

NA: Not Applicable; **N:** no; **Y:** yes; **y:** Years; **m: months; NI:** Not Informed.

Table 1S Clinical features described on the literature. (continuation)

	Qorri et al (2002)	Rombout et al (2004)	Su et al (2005)	Babic et al (2007)
**PATIENTS**	1	1	6	1
**Proximal 19q breakpoint described by authors**	**46,XX,dir dup(19)(q13.1q13.3)dn.ish dir dup(19)(q13.1q13.3)(wpc19+)**	**46,XX,der(22)t(19;22)(q13.3;p13)**	**der(9)t(9;19)(p24.1;q13.4)**	**46,XY,der(21)t(19;21)(q11;p13)mat**
de novo	Y	Y	N	N
**Sex**	female	female	3 males and 3 females	male
**Age**	2y3m	-	2 adults and 4 children	-
**Method of detection**	FISH	FISH	FISH	M-FISH
**Short stature**	N	NA	NI	NA
**IUGR/Growth delay**	N/N	N	NI	N
**FACIAL FEATURES**				
**Ocular hypertelorism**	NI	NA	NI	NI
**Nasal root abnormalities**	N	Y	Y	Y
**Abnormal ears**	N	Y	NI	Y
**Downturned corners od the mouth**	N	NI	NI	NI
**Small teeth with dystrophic enamel**	N	NA	NI	NA

**Short neck**	N	Y	NI	Y
**HANDS AND FEET**				
**Bilateral clinodactyly**	N	Y	NI	NI
**NERVOUS SYSTEM**				
**Intellectual disability**	Y	NA	N(A)/Y(C)	NA
**Motor developmental delay**	Y	NA	Y(C)	NA
**Speech delay**	Y	NA	NI	NA
**Seizures**	N	NA	NI	NA
**Structural brain abnormalities**	NI	Y	NI	Y
**Hypoplasia of Corpus Callosum**	NI	N	NI	NI
**Hypotonia**	N	NA	Y(2)	NA
**OTHER**				
**Pulmonary infections**	N	NA	NI	NA
**Urinary tract infections**	N	NA	NI	NA

NA: Not Applicable; **N:** no; **Y:** yes; **y:** Years; **m: months; NI:** Not Informed.

	Sauter et al (2007)	Zung et al (2007)	Bralo et al (2008)
**PATIENTS**	1	1	1
**Proximal 19q breakpoint described by authors**	**der(6)t(6;19)(q?ter;q 13.42)**	**47,XY,dup(19)(q12→ q13.3)**	**dup(19)(q12q13.2)dn**
de novo	Y	N	Y
**Sex**	female	male	female
**Age**	2y7m	14y	1y4m
**Method of detection**	FISH/qPCR primer-jumping	M-FISH/CGH	array CGH
**Short stature**		NI	N
**IUGR/Growth delay**	N/Y	N/N	N/Y
**FACIAL FEATURES**			
**Ocular hypertelorism**	NI	NI	Y
**Nasal root abnormalities**	Y	NI	Y
**Abnormal ears**	Y	Y	Y
**Downturned corners od the mouth**	NI	NI	Y
**Small teeth with dystrophic enamel**	NI	NI	NI

**Short neck**	Y	NI	Y
**HANDS AND FEET**			
**Bilateral clinodactyly**	NI	NI	NI
**NERVOUS SYSTEM**			
**Intellectual disability**	Y	Y	Y
**Motor developmental delay**	Y	Y	Y
**Speech delay**	Y	Y	Y
**Seizures**	NI	Y	NI
**Structural brain abnormalities**	Y	Y	Y
**Hypoplasia of Corpus Callosum**	Y	Y	Y
**Hypotonia**	Y	N	Y
**OTHER**			
**Pulmonary infections**	NI	N	NI
**Urinary tract infections**	NI	N	NI

NA: Not Applicable; **N:** no; **Y:** yes; **y:** Years; **m: months; NI:** Not Informed.

Table 2S Characterization of the common 0.4 Mb region shared by the seven patients analysed by array.

	Symbol	Band	start (hg19)	stop (hg19)
**Coding Genes**				
alpha-1-B glycoprotein	A1BG	19q13.4	58346806	58353499
A1BG antisense RNA 1	A1BG-AS1	19q13.4	58351970	58355183
zinc finger protein 497	ZNF497	19q13.43	58354357	58362848
zinc finger protein 837	ZNF837	19q13.43	58367623	58381022
ribosomal protein S5	RPS5	19q13.4	58387269	58394804
uncharacterized LOC646862	LOC646862	19q13.43	58395684	58397079
hepatocellular carcinoma-associated antigen HCA25a	LOC100506634		58403941	58408466
zinc finger protein 584	ZNF584	19q13.43	58407359	58418327
zinc finger protein 132	ZNF132	19q13.4	58432814	58440222
zinc finger protein 324B	ZNF324B	19q13.43	58451604	58457832
zinc finger protein 324	ZNF324	19q13.43	58457652	58473578
**zinc finger protein 446**	**ZNF446**	**19q13.43**	**58475878**	**58489533**
solute carrier family 27 (fatty acid transporter), member 5	SLC27A5	19q13.43	58498333	58512065
zinc finger and BTB domain containing 45	ZBTB45	19q13.43	58513530	58520478
tripartite motif containing 28	TRIM28	19q13.4	58544469	58550715
charged multivesicular body protein 2A	CHMP2A	19q	58551566	58555128
hCG_2045958	UBE2M	19q13.43	58555712	58558976
myeloid zinc finger 1	MZF1	19q13.4	58561917	58573760
**Pseudogenes**				
**zinc finger protein 446 pseudogene**	**LOC100419840**		**58358049**	**58359239**
zinc finger protein 250 pseudogene	LOC100419848		58444928	58445479
CENPBD1 pseudogene 1	CENPBD1P1	19q13.43	58575399	58584395
ribosomal protein L23a pseudogene 79	RPL23AP79	19q13.4	58598970	58599435
**MicroRNAs**				

**microRNA 4754**	**MIR4754**		**58386770**	**58386858**
microRNA mir-6807	MIR6807		58550285	58550376
**Uncharacterized Loci**				
uncharacterized LOC100131691	AC016629.8	19q13.43	58559186	58574797
uncharacterized LOC101929177	LOC101929177		58588048	58590925

REFERENCES

1. Lange M, Alfi OS. Trisomy 19 q. Ann Genet 1976: 19(1): 17-21.
2. Schmid W. Trisomy for the distal third of the long arm of chromosome 19 in brother and sister. Hum Genet 1979: 46(3): 263-270.
3. Zonana J, Brown MG, Magenis RE. Distal 19q duplication. Hum Genet 1982: 60(3): 267-270.
4. Rivas F, Garcia-Cruz D, Rivera H, Plascencia ML, Gonzalez RM, Cantu JM. 19q distal trisomy due to a de novo (19;22)(q13.2;p11) translocation. Ann Genet 1985: 28(2): 113-115.
5. Boyd E, Grass FS, Parke JC, Knutson K, Stevenson RE. Duplication of distal 19q: clinical report and review. Am J Med Genet 1992: 42(3): 326-330.
6. Quack B, Van Roy N, Verschraegen-Spae MR, Klein F. Interstitial deletion and ring chromosome derived from 19q. Proximal 19q trisomy phenotype. Ann Genet 1992: 35(4): 241-244.
7. Trautmann U, Pfeiffer RA, Seufert-Satomi U, Tietze HU. Simultaneous de novo interstitial deletion of 16q21 and intercalary duplication of 19q in a retarded infant with minor dysmorphic features. J Med Genet 1993: 30(4): 330-331.
8. Valerio D, Lavorgna F, Scalona M, Conte A. A new case of partial trisomy 19q (q13.2-->qter) owing to an unusual maternal translocation. J Med Genet 1993: 30(8): 697-699.
9. James C, Jauch A, Robson L, Watson N, Smith A. A 3 1/2 year old girl with distal trisomy 19q defined by FISH. J Med Genet 1996: 33(9): 795-797.
10. Cotter PD, McCurdy LD, Gershin IF, Babu A, Willner JP, Desnick RJ. Prenatal detection and molecular characterization of a de novo duplication of the distal long arm of chromosome 19. Am J Med Genet 1997: 71(3): 325-328.
11. Bhat M, Morrison PJ, Getty A, McManus D, Tubman R, Nevin NC. First clinical case of small de novo duplication of 19q (13.3-13.4) confirmed by FISH. Am J Med Genet 2000: 91(3): 201-203.
12. Tercanli S, Hosli I, Berlinger A, Beyer R, Achermann J, Holzgreve W. Prenatal diagnosis of a partial trisomy 19q. Prenat Diagn 2000: 20(8): 663-665.
13. Qorri M, Oei P, Dockery H, McGaughran J. A rare case of a de novo dup(19q) associated with a mild phenotype. J Med Genet 2002: 39(10): E61.
14. Rombout S, Sartenaer D, Parmentier B, Dugauquier C, Gillerot Y. A rare case of de novo distal 19q trisomy prenatally diagnosed. Prenat Diagn 2004: 24(10): 822-827.
15. Su PH, Kuo PL, Chen SJ, Huang SC, Chen JY, Hung HM. Six cases of deletion 9p24 and trisomy 19q13.4 inherited from a familial balanced translocation. J Formos Med Assoc 2005: 104(7): 525-530.
16. Babic I, Brajenovic-Milic B, Petrovic O, Mustac E, Kapovic M. Prenatal diagnosis of complete trisomy 19q. Prenatal Diagnosis 2007: 27: 644-647.
17. Sauter SM, Bohm D, Bartels I et al. Partial trisomy of distal 19q detected by quantitative real-time PCR and FISH in a girl with mild facial dysmorphism, hypotonia and developmental delay. Am J Med Genet A 2007: 143A(10): 1091-1099.
18. Zung A, Rienstein S, Rosensaft J, Aviram-Goldring A, Zadik Z. Proximal 19q trisomy: a new syndrome of morbid obesity and mental retardation. Horm Res 2007: 67(3): 105-110.
19. Palomares Bralo M, Delicado A, Lapunzina P et al. Direct tandem duplication in chromosome 19q characterized by array CGH. Eur J Med Genet 2008: 51(3): 257-263.
